# Probiotic Modulation in Aging: Strain-Specific Geroprotective Effects in *Caenorhabditis elegans*

**DOI:** 10.3390/ijms262211205

**Published:** 2025-11-20

**Authors:** Barbara Sciandrone, Diletta Francesca Squarzanti, Patrizia Malfa, Maria Elena Regonesi

**Affiliations:** 1 Department of Biotechnology and Biosciences BtBs, University of Milano-Bicocca, Piazza della Scienza 2, 20126 Milan, Italy; barbara.sciandrone@unimib.it; 2 SynBalance Srl, 21040 Origgio, Italyp.malfa@synbalance.care (P.M.); 3 Milan Center of Neuroscience (NeuroMI), 20126 Milan, Italy

**Keywords:** aging, *Caenorhabditis elegans*, geroprotector, gut-microbiota, probiotics

## Abstract

Elderly individuals are more vulnerable to disease due to their increased frailty. Emerging evidence highlights the potential of probiotics as geroprotective agents by maintaining gut health and modulating key physiological processes involved in aging, such as inflammation, cognitive functions, and metabolism. Here, we investigated the geroprotective potential of four probiotic strains (*Lacticaseibacillus paracasei* LPC1114, *Limosilactobacillus reuteri* PBS072, *Bifidobacterium breve* BB077, and *Bifidobacterium animalis* subsp. *lactis* BL050) using *Caenorhabditis elegans* as an aging model. Mid-life healthspan parameters were assessed, including lifespan, motility, ROS levels, lipofuscin accumulation, and cognitive capabilities. The probiotics exhibited strain-specific effects. *L. reuteri* PBS072 and *B. lactis* BL050 significantly increased locomotion by 20% and decreased ROS levels by 70% and 30% respectively, suggesting enhanced oxidative stress response and neuromuscular maintenance. *B. breve* BB077, *L. paracasei* LPC1114, and *L. reuteri* PBS072 enhanced associative learning performance, whereas *B. lactis* BL050 improved chemotactic response. Notably, only *L. paracasei* LPC1114 and *L. reuteri* PBS072 extended the maximum lifespan by 4 and 5 days, respectively, an effect mediated by the longevity-related genes *skn1*, *sir2.1*, and *daf16*. Our findings highlight the multifaceted, strain-specific geroprotective properties of probiotics and support their potential as microbiome-based interventions to promote healthy aging.

## 1. Introduction

During the aging process, many physiological changes occur, including increased oxidative stress and declines in metabolic, immunological, and cognitive functions. As a result, the prevalence of frailty and chronic diseases is increasing in elderly individuals [1,2,3,4]. Moreover, the 2024 WHO report shows that the number of people aged 60 and over is expected to double in the next 30 years [5] and, therefore, a growing field in the biology of healthy aging is focused on counteracting aging-related changes using geroprotector agents [6,7]. Geroprotectors are emerging agents with the capability of slowing down the aging process by minimizing cellular physiological perturbations, aiming to improve quality of life and to prevent age-related disorders [8,9,10,11,12]. In this context, increasing evidence over the past decade suggests that significant modifications of gut microbiota during aging are correlated with the development of aging-related conditions [9,13]. In particular, dysbiosis (i.e., alteration in gut microbiota composition and function) has been associated with several pathological conditions, including obesity, type 2 diabetes, inflammation, cardiovascular, and neurodegenerative diseases [14]. Therefore, maintaining a homeostasis in host-microbiota interaction is crucial to counteract inflammation, preserve intestinal permeability, and ameliorate both cognitive performance and muscle function [15,16,17,18]. The gut microbiota can be effectively and stably modulated through dietary interventions using different health-promoting components. Among these, probiotics exert multiple beneficial effects on host health, including age-related processes. In fact, the probiotic modulation of the gut microbiota is a valuable approach for the prevention and potential mitigation of age-related disorders [19]. In the FAO/WHO report on food and nutrition [20], probiotics are defined as “live microorganisms which, when consumed in adequate amounts, confer a health effect on the host” [21]. The potential role of probiotics in promoting longevity was first proposed in the early 20th century by Elie Metchnikoff, who observed a positive correlation between the consumption of *Lactobacillus bulgaricus*, contained in yogurt, and increased longevity in the Bulgarian population [22]. Historically, this hypothesis was further supported by the identification of good health-associated bacteria species in centenarians aged 105–109 years [23]. Finally, recent studies have demonstrated that gut microbiota modulation through probiotics in the elderly can improve immune response, cognitive function, and reduce body fat, metabolic syndromes, cardiovascular risk factors, and insulin resistance markers [24,25].

Lactic acid bacteria (LAB), especially *Lactobacillus* and *Bifidobacterium,* are two genera present in human microbiota and represent the most studied probiotics for their species- and strain-specific beneficial effects [26,27] and geroprotective properties [9,24,28]. Their benefits on health were evaluated and confirmed using different preclinical models, including mice, flies, and nematodes [29,30,31,32,33,34,35]. Among these models, *Caenorhabditis elegans*, a small nematode, provides several advantages for the study of age and host-microbiota interactions, including high experimental reproducibility, low maintenance costs, large size, short lifespan (~2–3 weeks), and no ethical constraints. Furthermore, *C. elegans* is a bacterivore organism normally maintained in axenic laboratory conditions using *Escherichia coli* OP50 as a food source, which prevents the formation of a complex intestinal microbiota [36]. This unique feature allows precise control of its gut microbiota through dietary manipulation and facilitates the evaluation of host-probiotic interactions and their physiological effects. The gnotobiotic nature of *C. elegans* makes it suitable for high-throughput screenings in a whole-organism, enabling the assessment of physiological responses across multiple tissues [37]. By monitoring key phenotypic traits such as lifespan, locomotion, reactive oxygen species (ROS) production, and cognitive-like functions, it is possible to evaluate the potential anti-aging impact of specific probiotic strains both in a controlled and efficient manner.

Taking advantage of the useful features of this model, we assessed the geroprotective effects of two LAB (*Lacticaseibacillus paracasei* LPC1114 and *Limosilactobacillus reuteri* PBS072) and two *Bifidobacteria* (*Bifidobacterium breve* BB077 and *Bifidobacterium animalis* subsp. *lactis* BL050) strains. These selected strains have been previously characterized for their probiotic activity [38,39,40,41], and their anti-inflammatory properties have been demonstrated both in vitro and in clinical studies. In particular, they have been shown to reduce proinflammatory cytokine levels and to alleviate symptoms associated with the cold, allergic, and urogenital diseases [39,40,42,43,44]. Additionally, *L. reuteri* PBS072 and *B. breve* BB077 have been shown to enhance serotonin production and to improve cognitive function, sleep quality, and mood under stress conditions, as demonstrated in three different clinical trials [45,46,47].

By assessing key healthspan and cognitive parameters, we aimed to uncover strain-specific effects on *C. elegans* aging-related traits. Our findings reveal that these probiotics exert distinct and multifaceted effects on neuromuscular function, oxidative stress response, cognitive performance, and, in some cases, lifespan extension. These effects seem to involve conserved signaling pathways related to immunity, stress resistance, and metabolism. Overall, our results support the potential of microbiome-based strategies to promote healthy aging and underline the importance of mechanistic insights into strain-specific probiotic actions.

## 2. Results

### 2.1. The Administration of Lactobacillus and Bifidobacterium spp. Ameliorates Phenotypic Aging Parameters in a Strain-Specific Manner

To evaluate the geroprotective effects of probiotics on healthspan parameters, synchronized *C. elegans* N2 nematodes were grown on *E. coli* OP50 until day-1 adulthood, then washed and transferred to NGM plates seeded with living *L. paracasei* LPC1114, *L. reuteri* PBS072, *B. breve* BB077, and *B. lactis* BL050 probiotic strains. Healthspan parameters, including lifespan, locomotion, total ROS levels, and lipofuscin accumulation data, were assessed on day 11th of adulthood and compared to control worms continuously fed with *E. coli* OP50.

**Figure 1:**
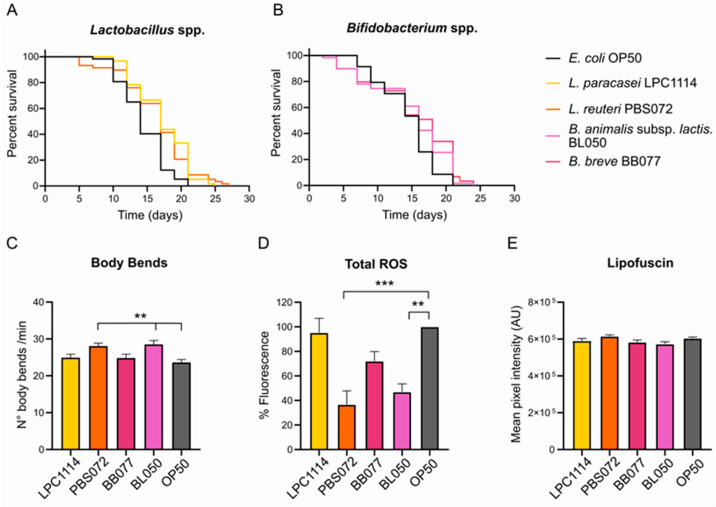
Effects of probiotics treatment on *C. elegans* healthspan parameters. (**A**,**B**). Lifespan assay of *C. elegans* population treated with LAB (**A**) and *Bifidobacteria* (**B**) strains. Probiotics were administered to a population of 60 1-day adult nematodes every two days until the whole population died. The representative Kaplan–Meier graphs with log-rank (Mantel–Cox) test were reported and represent the survival of the populations. (**C**–**E**) Healthspan parameters on day 11 of adulthood of *C. elegans* populations fed with probiotic strains. (**C**) Body bends in one minute. Data reported represents mean ± s.e.m. of 60 nematodes from three independent experiments. (**D**) Total ROS in *C. elegans* whole organisms. ROS signals were obtained from 20 living nematodes using the fluorescent probe DCF-DA and expressed as a percentage with respect to the control. Data reported represent mean ± s.e.m. of three independent experiments. (**E**) Lipofuscin content. Lipofuscin was measured by assessing autofluorescence by confocal analysis. Data reported represent mean ± s.e.m. of 30 nematodes that are derived from three independent biological experiments. An ordinary one-way ANOVA with Dunnet’s multiple comparison test was performed for the statistical analysis. ** *p* < 0.01, *** *p* < 0.001.

As shown in Figure 1A,B and Table 1, the survival curves of *C. elegans* grown on the different probiotic strains showed that the probiotic diets did not alter the median lifespan compared to the control. However, a significant increase in maximum lifespan was observed in worms fed with LAB strains, suggesting a targeted effect in aged worms. Specifically, populations fed with *L. paracasei* LPC1114 and *L. reuteri* PBS072 lived 4 and 5 days longer than the control group, respectively.

Additional healthspan parameters were assessed on day 11 of adulthood, including locomotion, total intracellular ROS levels, and lipofuscin accumulation. Among all probiotic treatments, only *L. reuteri* PBS072 and *B. lactis* BL050 induced statistically significant changes. Administration of these strains led to an approximately 20% increase in body bends (Figure 1C) and a significant reduction in total ROS levels of about 70% and 30%, respectively (Figure 1D). In contrast, none of the probiotic strains, including *L. reuteri* PBS072 and *B. lactis* BL050, produced significant changes in lipofuscin accumulation compared to the control (Figure 1E and Figure S1).

To confirm the presence of the administered probiotics in the gut, colony-forming units (CFUs) were quantified in aged nematodes (11th day of adulthood). Although *E. coli* OP50 exhibited a CFU count up to 10^5^-fold higher, the probiotic strains showed comparable levels of gut colonization (Table 2). This indicates that the observed beneficial effects are not attributable to differences in bacterial load within the gut.

These findings suggest that the administration of LAB and *Bifidobacteria* spp. improves phenotypic aging markers in a strain-specific manner.

### 2.2. Probiotic Diets Do Not Affect Neuromuscular Trasmission but Enhance Cognitive Functions in Aged C. elegans

**Figure 2:**
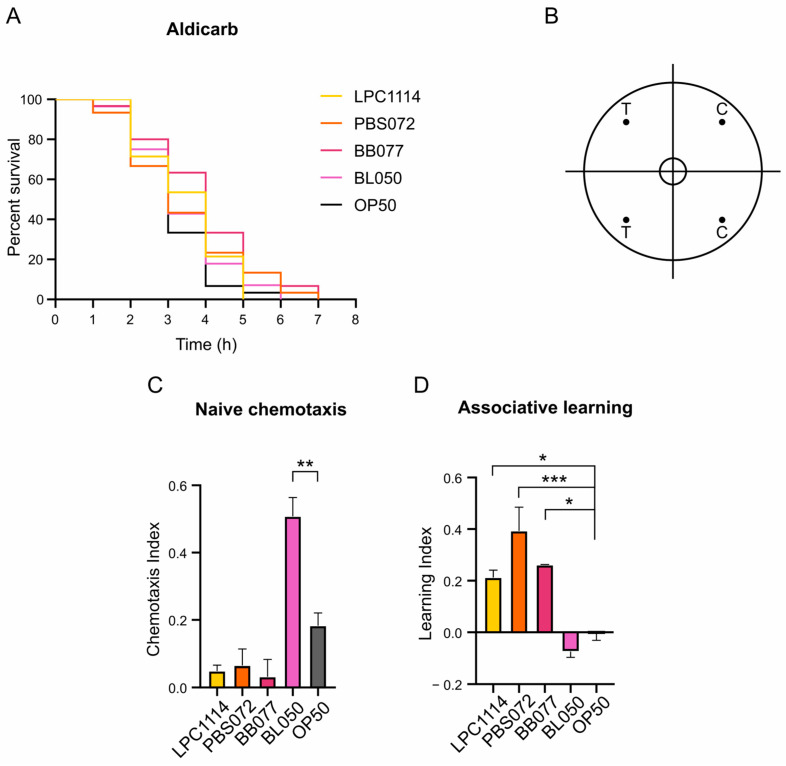
Effects of probiotics treatment on *C. elegans* neuronal functions at the 11th day of adulthood treated with LAB and *Bifidobacteria* probiotic strains. (**A**) Synaptic impairment was assessed through Aldicarb assay. On day 11 of lifespan, 30 nematodes were treated with 1 mM of Aldicarb. Representative Kaplan–Meier graphs with log-rank (Mantel–Cox) test of the survival of the populations were reported. No significant differences were observed. (**B**) Representative chemotaxis test plate. T = test quadrants with 10% butanone; C = control quadrants with EtOH. Dots represent the point in which butanone or EtOH were dropped. (**C**,**D**). Naïve chemotaxis and associative learning graphs. Chemotaxis and learning indices were calculated as described in Materials and Methods section. The experiment was performed using at least 100 nematodes. Data represents the mean ± s.e.m. of three independent experiments. Ordinary one-way ANOVA with Dunnet’s multiple comparison test was performed for statistical analysis. * *p* < 0.05, ** *p* < 0.01, *** *p* < 0.001.

The age-related decline in motility observed in *C. elegans* is partly attributed to the deterioration of motor neurons [48]. To determine whether the observed increase in motility was associated with enhanced synaptic transmission in motor neurons, nematodes fed with probiotic strains were subjected to Aldicarb sensitivity assay on day 11 of adulthood. Aldicarb acts by inhibiting the acetylcholinesterase enzyme, leading to the accumulation of acetylcholine at the neuromuscular junction and ultimately causing paralysis [49]. Differences in the time to paralysis can thus indicate alterations in neuromuscular signaling. Following administration of 1 mM Aldicarb, no significant differences in paralysis onset were observed among the groups (Figure 2A), indicating that probiotic diets do not affect neuromuscular transmission.

Beyond motor function, aging also impairs sensory perception due to progressive neuronal functions decline. In *C. elegans,* odor sensitivity decreased with age as a consequence of sensory neuronal circuits deterioration [50]. The nematode can distinguish between a plethora of attractive and repellent odorants through its 24 chemosensory neurons [51,52]. To evaluate whether probiotics supplementation supports sensory neuronal function during aging, chemotaxis assays were performed. Butanone, recognized by *C. elegans* as an attractive stimulus, and ethanol, used as a neutral control, were spotted on opposite sides of the chemotaxis plate (Figure 2B). On the 11th day of adulthood, nematodes were extensively washed and dropped in the center of a plate. After one hour, the naïve chemotaxis index (Ci_naïve_) was calculated, and the results showed that nematodes fed with *B. lactis* BL050 exhibited a two-fold increase in attraction to butanone compared to controls fed with *E. coli* OP50 (Figure 2C). In contrast, worms treated with the *L. paracasei* LPC1114, *L. reuteri* PBS072, or *B. breve* BB077 showed a slight but non-significant reduction in Ci_naïve_ (Figure 2C).

Finally, the ability of probiotic strains to enhance learning was evaluated in the same population. *C. elegans* can perform associative learning by linking an odor with food presence [53]. After washing with M9 buffer and one hour of starvation, worms were exposed to butanone to induce conditioning. Following an additional wash, chemotaxis index (Ci) after conditioning was measured (Table 3) and compared with the Ci_naïve_ to calculate the learning index (Li; see Materials and Methods section for formula details). Otherwise to the Ci_naïve_ results, *L. paracasei* LPC1114, *L. reuteri* PBS072, and *B. breve* BB077 treatment led to a significant increase in Li compared to the control (Figure 2D and Table 3), whereas *B. lactis* BL050 showed no improvement in Li.

These findings demonstrate that *L. paracasei* LPC1114, *L. reuteri* PBS072, *B. breve* BB077, and *B. lactis* BL050 can ameliorate neuronal plasticity in aging nematodes, although different mechanisms, supporting their strain-specific geroprotective effects on the nervous system.

### 2.3. Probiotic Diets Differently Increase Progeny Production

**Figure 3:**
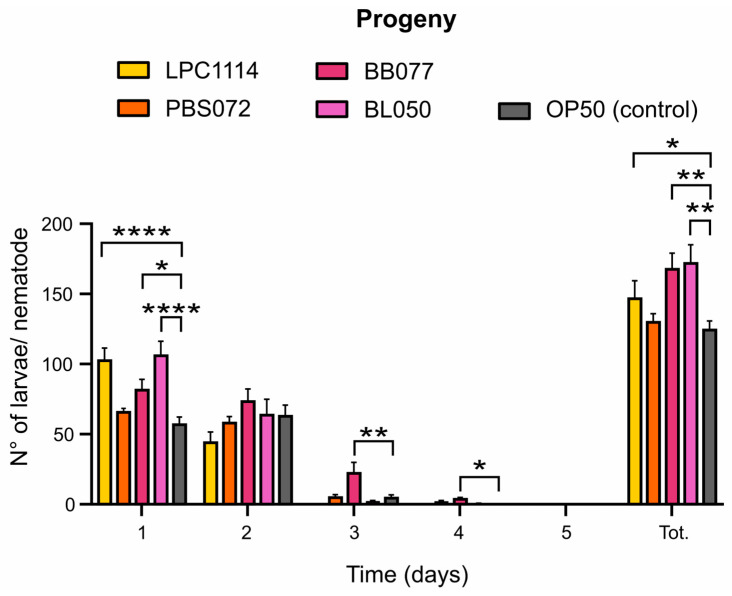
Effects of probiotics on *C. elegans* progeny. The mean ± s.e.m. of progeny from 10 nematodes was reported. Ordinary one-way ANOVA with Dunnet’s multiple comparison test was performed for statistical analysis. * *p* < 0.05, ** *p* < 0.01, **** *p* < 0.0001.

In *C. elegans*, the fertile period is about five days starting from the first day of adulthood, with a peak in egg-laying on day one followed by a rapid decline [36]. Aging reduces offspring production from the second fertility day, but longevity-promoting agents can influence both total progeny and the egg-laying deposition [54]. To assess the impact of probiotic supplementation on fertility, the daily progeny deposited by a single nematode was quantified over five days (Figure 3). The results showed that the administration of the strains *L. paracasei* LPC1114, *B. breve* BB077, and *B. lactis* BL050 led to an increase in total progeny of 18%, 39%, and 42%, respectively; conversely, *L. reuteri* PBS072 showed no significant differences compared to the control. Notably, *L. paracasei* LPC1114 and *B. lactis* BL050 stimulated egg deposition primarily on day one. Interestingly, *B. breve* BB077 significantly altered the deposition curve, with a marked increase in egg-laying on days two and three, 5-fold and 17-fold higher compared to the control, respectively. This clearly demonstrates that *B. breve* BB077 is the only strain capable of exerting an anti-senescence effect on the laying curve by extending fertility beyond the initial reproductive burst.

### 2.4. Identification of Genes Involved in Lifespan Extension

**Figure 4:**
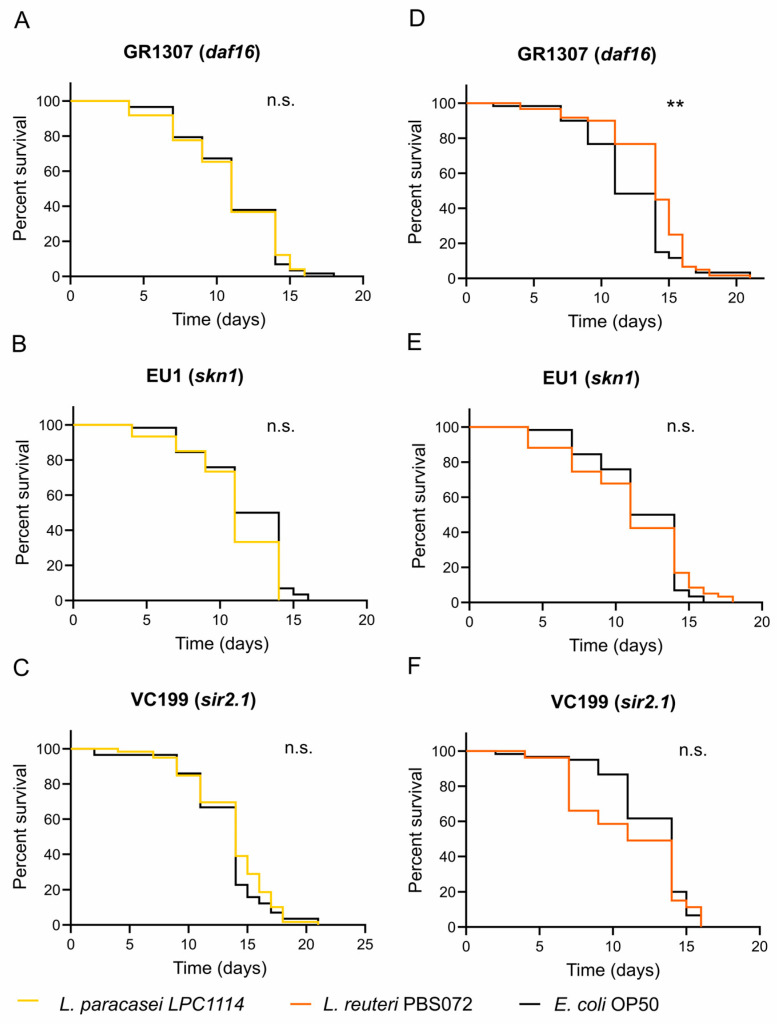
Lifespan assay of *C. elegans* mutants treated with LAB strains. *L. paracasei* LPC1114 (**A**–**C**) and *L. reuteri* PBS072 (**D**–**F**) were administered to a 1-day adult *C. elegans* GR1307 (Δ*daf16*), EU1 (Δ*skn1*), and VC199 (Δ*sir-2.1*) mutants every two days until the whole population died. The representative Kaplan–Meier graphs with log-rank (Mantel–Cox) test were reported and represent the survival of the populations. ** *p* < 0.01. n.s. not significant.

As mentioned in the introduction, the lifespan extension and geroprotective effects of probiotics may involve the regulation of evolutionarily conserved pathways related to innate immunity, energy metabolism, and cellular stress response. These pathways are functionally interconnected and converge on two key transcriptional factors: DAF16 (FOXO human ortholog) and SKN1 (Nrf2 human ortholog) [9,37]. To identify which pathways are involved in the lifespan extension observed with LAB strains (Figure 1A and Table 1), lifespan assays were performed using the mutant strains GR1307 (Δ*daf16*) and EU1 (Δ*skn1*) fed with *L. paracasei* LPC1114, *L. reuteri* PBS072, or *E. coli* OP50 (as a control) since the first day of adulthood. Worms fed with *L. paracasei* LPC1114 showed no significant differences in survival in both mutants (Figure 4A,B and Table 4) with respect to the control, indicating that both DAF16 and SKN1 are required for its pro-longevity effect. In contrast, worms fed with *L. reuteri* PBS072 showed an extended lifespan in the DAF16 mutant but not in the SKN1 mutant, suggesting that only SKN1 is involved in mediating its probiotic effect (Figure 4D,E and Table 4).

To further explore the molecular basis of these differential responses, we considered the role of SIR2.1, a NAD^+^-dependent deacetylase implicated in longevity and activated by different signals (such as dietary restriction) [55,56,57]. In our assays (Figure 4C,F and Table 4), neither LAB strains extended the lifespan in the VC199 strain (Δ*sir2.1*),indicating that SIR2.1 is essential for their pro-longevity activity, likely through distinct signaling mechanisms. Collectively, these data suggest that *L. paracasei* LPC1114 effects require the coordinated activity of DAF16, SKN1, and SIR2.1, whereas *L. reuteri* PBS072 acts primarily through SKN1 and SIR2.1.

## 3. Discussion

Aging is a multifactorial, physiological process characterized by the gradual deterioration of cellular functions, which increases the risk of pathological diseases in the elderly [1]. Although aging is a natural and inevitable aspect of life, its progression and impact on health can vary significantly among individuals, suggesting the involvement of other factors beyond genetics. Among these factors, the gut microbiota composition has emerged as a key determinant of healthy aging and is now widely recognized as one of the main modulators of age-related physiological decline [58,59,60,61]. In fact, it has been reported that microbiota in centenarians contains specific beneficial bacterial species that are positively correlated with healthy aging [62,63]. These findings prompted scientists to further investigate the mechanisms underlying the beneficial probiotics’ effects on aging, using model organisms. Several findings indicate that probiotics can ameliorate healthspan and, in some cases, extend the lifespan by modulating specific biological pathways [9,37].

In this work, we used the well-characterized *C. elegans* model organism for studying the geroprotective effects of four probiotic strains: *L. paracasei* LPC1114, *L. reuteri* PBS072, *B. breve* BB077, and *B. lactis* BL050. *C. elegans* is a well-established model organism for aging research due to its several advantageous features, including short lifespan (2–3 weeks), fully sequenced genome, and highly conserved aging-related pathways [64,65,66,67]. We exploited these characteristics to evaluate multiple phenotypic parameters in nematode populations fed with a single living probiotic strain until mid-life (11th day of adulthood, except for lifespan assay). This time point corresponds to a stage when age-related physiological decline becomes evident, allowing for a comparison of aging-associated traits [68,69]. Starting with the general healthspan parameters, i.e., lifespan, locomotion, total ROS levels, and lipofuscin accumulation, we observed strain-specific improvements in the phenotypic traits during aging. In particular, *L. reuteri* PBS072 and *B. lactis* BL050 administration ameliorated movement and reduced total ROS content, but only *L. reuteri* PBS072 significantly extended lifespan. Otherwise, *L. paracasei* LPC1114 increased maximum lifespan, but did not ameliorate the other healthspan parameters, while *B. breve* BB077 showed no beneficial effects on any of the assessed parameters. Notably, the observed increase in the body bends following probiotics administration does not appear to be associated with enhanced synaptic transmission. This is supported by the lack of differences in survival curves after Aldicarb treatment, suggesting that *L. reuteri* PBS072 and *B. lactis* BL050 likely promote locomotion through enhanced muscle function rather than neuronal modulation. The lack of effect of these probiotic strains on Aldicarb-induced paralysis, with respect to *E. coli* OP50 used as a control, may reflect their limited influence on acetylcholine signaling at the neuromuscular junction. Aldicarb induces paralysis by inhibiting acetylcholinesterase, leading to acetylcholine accumulation and muscle hypercontraction. Probiotics that primarily modulate oxidative stress or overall healthspan may not significantly affect this pathway. Furthermore, strain-specific differences in metabolite production might be insufficient to alter Aldicarb sensitivity.

On the other hand, we observed improvements in cognitive functions, indicating that the nervous system may also benefit from specific probiotic geroprotection. Specifically, we evaluated both the naïve chemotaxis (Ci_naïve_) and associative learning (Li) indexes using butanone 10%. The treatment with both LAB and *B. breve* BB077 did not alter the Ci_naïve_, while *B. lactis* BL050 administration led to a two-fold increase in attraction to butanone compared to control nematodes fed with *E. coli* OP50. A similar probiotic-enhanced chemotaxis activity was previously reported in aged *C. elegans* fed with *Limosilactobacillus reuteri* (formerly *Lactobacillus reuteri*), where an increased naïve chemotaxis response to diacetyl was observed in comparison with *E. coli*-fed nematodes. This enhancement was attributed to upregulated expression of the diacetyl receptor ODR-10, which appears to be regulated by DAF-16 transcriptional factor [70]. Butanone is one of the five attractive volatile odorants sensed by the paired Amphid wing “C” (AWC) neurons [71], and its recognition is specifically dependent on the transmembrane guanylyl cyclase ODR-1 localized in the sensory cilia of AWC [72]. Although its regulation is not yet fully understood, it is possible to hypothesize that *B. lactis* BL050 administration may enhance *odr-1* expression, as observed for ODR-10 receptor.

Moreover, previous studies have shown that the pre-exposure to butanone in combination with food (a process known as conditioning) induced a specific form of behavioral plasticity, called butanone enhancement, characterized by increased chemotaxis toward butanone following conditioning [53]. This represents an example of sensory integration, in which the combination of two stimuli, odor and food, triggers a plastic behavioral response like associative learning. In this context, our results showed that feeding *C. elegans* with *L. paracasei* LPC1114, *L. reuteri* PBS072, or *B. breve* BB077, but not with *B. lactis* BL050, enhances the associative learning index (Li) after conditioning in the presence of food with respect to the worms fed with *E. coli* OP50. It is important to note, however, that *B. lactis* BL050 Ci after conditioning has the highest index, resulting in a comparatively lower Li. These findings support the earlier hypothesis that the ODR-1 expression may also be upregulated in *B. lactis* BL050-fed nematodes, leading to a stronger attraction to butanone and thus a reduced capacity for further associative learning. Further investigations are needed to elucidate the underlying mechanism of this effect during *C. elegans* aging. Overall, our results highlight the strain-specific effects of tested probiotics on cognitive functions in *C. elegans* aging.

Then, we investigated the effects of the different probiotic strains on reproductive aging. Both *Bifidobacteria* strains and *L. paracasei* LPC1114 increased the total number of larvae produced; however, only *B. breve* BB077 significantly extended the egg-laying curve, resulting in a prolonged reproductive span. Aging in *C. elegans* is known to affect reproduction through various mechanisms [54], and serotonin plays a fundamental role in regulating egg-laying and oocyte quality [73]. The extended reproductive span observed with *B. breve* BB077 could be attributed to enhanced function of aged serotonin-producing neuron function and/or increased serotonin levels. This hypothesis is also supported by a previous work in which *B. breve* BB077 has been shown to increase serotonin production in *in vitro* cell lines [45].

Finally, we used *C. elegans* Δ*daf16*, Δ*skn1,* and Δ*sir-2.1* mutants to identify the molecular pathways involved in lifespan extension induced by *L. paracasei* LPC1114 and *L. reuteri* PBS072 strains. These genes play crucial roles in the signaling pathways that mediate lifespan extension induced by probiotic bacteria [9]. In *C. elegans*, DAF16 and SKN1 are homologous to mammalian FOXO and Nrf2 transcriptional factors, respectively, and both contribute to promoting *C. elegans* lifespan and healthspan through the insulin/IGF-1 (insulin-like growth factor) signaling (IIS) pathway [9,36]. Inhibition of the insulin/IGF-1 receptor activity leads to the nuclear accumulation of both SKN1 and DAF16 in intestinal cells, thereby activating downstream target genes involved in stress resistance and longevity [74]. *L. paracasei* LPC1114 failed to extend lifespan in both Δ*daf16* and Δ*skn1* mutants, indicating that these two key genes are required for its pro-longevity effect via the modulation of the IIS pathway. In addition, *L. paracasei* LPC1114 enhances nervous system plasticity, with no other major healthspan improvements observed. These two phenotypical outcomes may be functionally related, as gustatory and olfactory neurons contribute to lifespan extension through the IIS pathway [75]. In particular, increased neuronal DAF-16 activity modestly induced the expression of its target genes in other tissues (i.e., muscle and epidermis), resulting in a 5–20% increase in lifespan, even though mid-life motility remains unchanged [76,77].

On the other hand, *L. reuteri* PBS072 lifespan extension seems to be dependent on SKN1, but not on DAF16. SKN1 is known to mediate oxidative and xenobiotic stress response, regulate proteasome activity under stress conditions, and maintain cellular homeostasis under non-stress conditions [74]. Besides its regulation via the IIS pathway, SKN1 can be activated by phosphorylation through the p38 kinase PMK1 (the *C. elegans* homolog of human p38-MAP kinase) in response to inflammation and immune modulation, two hallmarks of immunosenescence [78,79]. We speculate that this mechanism could contribute to SKN1 activation by *L. reuteri* PBS072. SKN1 downstream gene targets regulate Phase I, II, and III detoxification pathways, contributing to both acute defense mechanisms and cellular homeostasis maintenance [74,80]. This dual function likely explains the observed reduction in ROS levels and the improvement in locomotion, consistent with studies linking reduced oxidative stress to enhanced motility in *C. elegans* [81,82,83].

SIR2.1, a member of the NAD+-dependent SIR2 deacetylases family, is a well-known regulator of lifespan in various organisms, including *C. elegans*, *Drosophila melanogaster*, and mammals [55]. SIR-2.1 can mediate lifespan extension through two main mechanisms. In the first, SIR2.1 promotes DAF16 nuclear translocation via interaction with the conserved acidic protein 14-3-3 under stress conditions [56], acting in parallel with the IIS pathway, both converging on the DAF16 transcriptional factor. This mechanism appears to be involved in *L. paracasei* LPC1114-mediated lifespan extension, as it fails to extend lifespan in SIR2.1, DAF16, and SKN1 *C. elegans* mutants. The second mechanism by which SIR2.1 can mediate lifespan extension is DAF16-independent [57,84]. It has been shown that resveratrol extends lifespan by mimicking dietary restriction in *C. elegans* through the involvement of both SIR2.1 and the energy-sensing AMP-activated protein kinase (AMPK), but not DAF-16 [57]. This mechanism seems to work in parallel with the SKN1 pathway to promote the lifespan extension observed with *L. reuteri* PBS072, as Δ*sir2.1 C. elegans* mutant hasn’t extended lifespan after *L. reuteri* PBS072 supplementation. While our lifespan assays in DAF-16, SKN-1, and SIR-2.1 mutants suggest a potential involvement of these key regulators in mediating the observed effects, we emphasize that these results are preliminary. Direct molecular evidence—such as qPCR analysis of downstream target genes or reporter assays—will be necessary to definitively clarify their involvement.

In conclusion, our results demonstrate that the probiotic strains *L. paracasei* LPC1114, *L. reuteri* PBS072, *B. breve* BB077, and *B. lactis* BL050 exert strain-specific geroprotective effects in the *C. elegans* model. While *L. paracasei* LPC1114 and *L. reuteri* PBS072 showed both healthspan and lifespan enhancement, *B. breve* BB077 and *B. lactis*. BL050 significantly improved healthspan parameters without extending lifespan. These findings underline the importance of distinguishing between healthspan and lifespan in the evaluation of probiotic efficacy. The observed difference in the outcomes could be attributed to the distinct bioactive compounds and metabolites produced by each strain, which may act as signaling molecules, influencing host physiology through specific molecular pathways. Further metabolomic and transcriptomic analyses will be essential to elucidate these mechanisms and identify the involved compounds. Importantly, although certain strains did not promote longevity under basal conditions, their positive impact on health-related functions suggests a potential geroprotective role under stress conditions, such as oxidative stress. Future investigations will be necessary to clarify this observation.

*C. elegans* proves to be a robust and versatile model for the screening of probiotic geroprotective properties. Its bacterivorous nature allows for direct dietary modulation of the gut microbiota, enabling the evaluation of individual probiotic strains in vivo, yet within the complexity of a multicellular organism. This aspect makes *C. elegans* a useful organism to evaluate tissue-specific responses and the modulation of the gut-brain axis upon probiotic administration, both of which are crucial aspects in the development of effective probiotic-based interventions aimed at promoting healthy aging.

Overall, our findings contribute to the growing evidence supporting the use of specific probiotics as modulators of host aging and healthspan and lay the basis for future studies aiming to translate these effects into higher organisms and, eventually, clinical applications.

## 4. Materials and Methods

### 4.1. Caenorhabditis elegans Strains and Maintenance

The *C. elegans* wild-type N2 (Bristol), GR1307 (Δ*daf16*), EU1 (Δ*skn1*), and VC199 (Δ*sir-2.1*) strains used in this work were procured from the *Caenorhabditis* Genetics Center, University of Minnesota (CGC, University of Minnesota, Minneapolis, MN, USA). All the strains were maintained on Nematode Growth Medium (NGM; 50 mM NaCl, 2.5 g/L peptone, 17 g/L agar; 1 mM CaCl_2_, 1 mM MgSO_4_, 5 μg/mL cholesterol in ethanol) plates, at 20 °C and seeded with live *E. coli* OP50 at OD_600_ = 1 as a food source. Synchronized adult populations were obtained by placing gravid worms on NGM plates seeded with *E. coli* OP50 and allowed to lay eggs for 16 h at 20 °C. Then, adult worms were sacrificed, and newly laid eggs were grown at 20 °C until they reached the 1-day adult stage. The synchronized adult populations obtained were then used to perform experiments.

### 4.2. Bacterial Strains and Culture Preparations

*Lacticaseibacillus paracasei* LPC1114 (DSM 34559), *Limosilactobacillus reuteri* PBS072 (DSM 25175), *Bifidobacterium breve* BB077 (LMG P-30157), and *Bifidobacterium animalis* subsp. *lactis* BL050 (DSM 25566) were provided by SynBalance S.r.l. (Origgio (VA), Italy) and cultured overnight in static condition, at 37 °C using the De Man, Rogosa, and Sharpe (MRS) broth, with the supplementation of 0.05% cysteine chlorohydrate for *Bifidobacteria* strains. The *E. coli* OP50 was grown in Luria Bertani broth overnight, at 37 °C with shaking. Then, strains were collected and washed with M9 buffer (22 mM KH_2_PO_4_, 42 mM Na_2_HPO_4_, 86 mM NaCl, 1 mM MgSO_4_) and resuspended at final OD_600_ = 1.

### 4.3. Lifespan Assay

Sixty synchronized nematodes were moved on NGM plates seeded with 200 μL of living bacterial strains at OD_600_ = 1. Every two days, living nematodes were scored and moved to new, fresh plates. Nematodes were considered dead if they did not respond to a gentle stimulation with the transfer pick. 5-Fluoro-2′-deoxyuridine (FUDR) 0.04 μM was added only in the first week to prevent eggs from hatching. The experiment was replicated at least three times, and median and maximum values were reported in the correlated table as mean ± s.d. Log-rank Mantel–Cox test of Kaplan-Meier survival curves was performed for the statistical analysis.

All subsequent experiments were conducted with animals at day 11 of the lifespan.

### 4.4. Colony-Forming Unit (CFU) Count

Probiotics CFU in aged worms was performed following the protocol described by Palominos et al., 2020 [85]. Briefly, 30 nematodes grown on probiotics strains or *E. coli* OP50 were picked up on day 11 of adulthood and washed at least three times with 1 mL of PBST. Then nematodes were resuspended in a final volume of 100 μL and disrupted mechanically using a micropestel. Nematode lysates were serially diluted and plated on MRS agar. For *Bifidobacteria* strains, MRS plates were supplemented with 0.05% cysteine chlorohydrate. Plates were incubated for 72 h at 37 °C in anaerobic condition and then the colonies were counted. The experiment was replicated at least three times, and mean ± s.d was reported.

### 4.5. Body Bends Assay

The motility of twenty nematodes was evaluated by counting the body bend numbers using the SteREO Discovery V12 microscope (Carl Zeiss Microscopy GmbH, Munich, Germany), as previously described [86]. The experiment was replicated three times and ordinary one-way ANOVA with Dunnett’s multiple comparison test was used for the statistical analysis.

### 4.6. ROS Mesurements

Total ROS content can be quantified in living nematodes using the 2,7-dichlorofluorescein diacetate (H_2_DCFH-DA; Sigma-Aldrich Co., St. Louis, MO, USA) permeable probe. Briefly, H_2_DCFH-DA is a probe that can easily permeate into the cells, and rapidly oxidize by ROS, producing the highly fluorescent DCF form [87]. Twenty nematodes were extensively washed with PBS to remove bacteria and moved to a black 96 well plate. Then, 50 µM of H_2_DCFH-DA probe was added and the fluorescence (485 nm excitation, 530 nm emission) was measured at 37 °C for 6 h using a multi-well fluorophotometer (Victor 3, PerkinElmer, Waltham, MA, USA). The experiment was replicated three times and the data were normalized with respect to *E. coli* OP50, used as a control, and reported as mean ± s.e.m. Ordinary one-way ANOVA with Dunnett’s multiple comparison test was performed.

### 4.7. Lipofuscin Accumulation

Thirty nematodes were assessed for lipofuscin accumulation. Briefly, nematodes were washed with PBS and immobilized using a solution of 5% glycerol and 1% NaN_3_ in PBS. Images were obtained using the Nikon confocal microscope system A1 (Nikon Europe B.V., Amstelveen, The Netherlands) and processed with Nikon NIS-Element AR 6.20.02 software. Lipofuscin quantification was performed using FITC (gain 500 ms) filterset (Nikon Europe B.V., Amstelveen, The Netherlands) and the mean pixel intensity (AU) was measured with Fiji (ImageJ 1.54g) software. Data reported represent mean ± s.e.m. of 30 nematodes that were derived from three independent biological experiments. An ordinary one-way ANOVA with Dunnett’s multiple comparison test was performed for the statistical analysis.

### 4.8. Aldicarb Assay

Thirty nematodes were transferred onto NGM plates supplemented with 1 mM Aldicarb, in the absence of bacteria, following the protocol described before [49]. Aldicarb acts by inhibiting the acetylcholinesterase enzyme, leading to the accumulation of acetylcholine at the neuromuscular junction and ultimately causing paralysis. Briefly, the nematode response to gentle stimulation with a worm picker was assessed every hour, until all worms were paralyzed. The experiment was repeated three times and statistical analysis was performed using the log-rank Mantel–Cox test on Kaplan–Meier survival curves.

### 4.9. Cognitive Tests

At least one hundred nematodes were assayed for cognitive tests, including naïve chemotaxis (Ci_naïve_) and associative learning (Li), following the protocol described by Heydarian et al., 2024 [88]. Briefly, after extensive washing with M9 buffer, nematodes were placed at the center of the chemotaxis plates (Figure 2B), where a drop of 1.5 μL of either 10% butanone (attractant) or 95% EtOH (control) solution was applied to the four corners of the plates. After 1 h, nematodes present in the two poles were counted to determine the Ci_naïve_, following the above formula.

$$Ci=\frac{n.{}^{\circ}\;worms\;on\;T-n.{}^{\circ}\;worms\;on\;C}{n.{}^{\circ}\;worms\;on\;T+C}$$

For the associative learning assay, after washing, nematodes were starved for 1 h in M9 buffer and then placed on a plate seeded with OP50 bacteria. For the conditioning, 2 μL of 10% butanone was applied to the inner side of the plate’s lid. Then, worms were washed with M9 and placed on the chemotaxis plates as described above. After 1 h of chemotaxis, the number of nematodes at the two poles was counted, and the conditioned chemotaxis index (Ci after *conditioning*) was calculated. The Li index was determined using the formula:

Li=Ci after conditioning−Ci naïve 

Data represents the mean ± s.e.m. of three independent experiments and ordinary one-way ANOVA with Dunnett’s multiple comparison test was used for the statistical analysis.

### 4.10. Fertility

The progeny of 1-day adult nematodes was evaluated by counting the number of larvae released during the first five days of adulthood. A single nematode was moved on an NGM petri dish seeded with living bacteria at OD_600_ = 1 and allowed to lay eggs. Every day for 5 days, the nematode was transferred to a fresh-seeded NGM plate. Then, the same plate was maintained at 20 °C overnight to get eggs hatching, and then the larvae were counted. The experiment was replicated ten times for each condition and an ordinary one-way ANOVA with Dunnett’s multiple comparison test was performed for the statistical analysis.

## Figures and Tables

**Table 1:** Lifespan parameters of *C. elegans* treated with LAB and *Bifidobacteria* probiotic strains. The table reports the median and maximum lifespan values obtained from the Kaplan–Meier graph (Figure 1) and expressed as the mean ± standard deviation from three independent experiments.

Strain	Median Lifespan (Days) ^1^	Maximum Lifespan (Days) ^2^	*p*-Value ^3^
LPC1114	15.5 ± 1.73	25 ± 0.00	0.0217
PBS072	15.5 ± 1.53	26.3 ± 2.08	0.0007
BB077	15.5 ± 1.91	26.5 ± 2.52	n.s.
BL050	15.2 ± 1.50	24.3 ± 2.52	n.s.
OP50	14.7 ± 1.15	21.0 ± 0.00	n.s.

^1^ Median lifespan: the day when 50% of nematodes used in the assay were live. Mean ± standard deviation was reported. ^2^ Maximum lifespan: the oldest age reached by the last surviving worm in each treatment. Mean ± standard deviation was reported. ^3^ The *p*-value was calculated using the log-rank test by comparing the probiotic treatment with the control. n.s.= non-significant.

**Table 2:** Colonization ability of probiotic strains in the nematode intestine at day 11 of adulthood. Colony Forming Unit (CFU) per mL was obtained from nematode lysate and mean ± s.d., from three replicates was reported.

Strain	CFU/mL
LPC1114	8.7 ± 2.9 × 10^1^
PBS072	11.7 ± 1.5 × 10^1^
BB077	5.3 ± 1.5 × 10^1^
BL050	22.3 ± 6.8 × 10^1^
OP50	16.5 ± 2.1 × 10^6^

**Table 3:** Chemotaxis index after butanone enhancement. The table presents the mean ± s.e.m. of three independent experiments.

Strain	Ci After Conditioning ^1^	*p*-Value ^2^
LPC1114	0.210 ± 0.042	n.s.
PBS072	0.404 ± 0.088	n.s.
BB077	0.465 ± 0.057	0.0266
BL050	0.537 ± 0.045	0.0103
OP50	0.166 ± 0.017	n.s.

^1^ Ci after conditioning is calculated as the naïve chemotaxis indices (see Materials and Methods section). ^2^ The *p*-value was calculated using the ordinary one-way ANOVA with Dunnet’s multiple comparison test. n.s.= non-significant.

**Table 4:** Lifespan parameters of *C. elegans* treated with LAB. The table presents the median and maximum days of the lifespan reported from the Kaplan–Meier graph (Figure 4) and expressed as the mean ± s.d. from three independent experiments.

*C. elegans* Mutant	Strain	Median Lifespan (Days) ^1^	Maximum Lifespan (Days) ^2^	*p*-Value ^3^
GR1307 (Δ*daf16*)	LPC1114	13.2 ± 1.75	17.5 ± 2.63	n.s.
	PBS072	14.0 ± 0.00	20.0 ± 1.50	0.0077
	OP50	11.0 ± 1.73	18.0 ± 2.08	
EU1 (Δ*skn1*)	LPC1114	12.0 ± 1.53	14.0 ± 2.31	n.s.
	PBS072	11.0 ± 0.58	18.0 ± 1.73	n.s.
	OP50	12.5 ± 2.57	16.0 ± 1.73	
VC199 (Δ*sir-2.1*)	LPC1114	14.0 ± 0.00	18.5 ± 3.56	n.s.
	PBS072	14.0 ± 2.45	21.5 ± 3.40	n.s.
	OP50	14.0 ± 1.00	18.0 ± 3.61	

^1^ Median lifespan: the day when 50% of nematodes used in the assay were live. Mean ± standard deviation was reported. ^2^ Maximum lifespan: the oldest age reached by the last surviving worm in each treatment. Mean ± standard deviation was reported. ^3^ The *p*-value was calculated using the log-rank test by comparing the probiotic treatment with the control. n.s.= non-significant.

## Data Availability

The original contributions presented in this study are included in the article/Supplementary Material. Further inquiries can be directed to the corresponding author.

## Supplementary Materials

The following supporting information can be downloaded at: https://www.mdpi.com/article/10.3390/ijms262211205/s1.
