# Supplementary material for: Probiotic Modulation in Aging: Strain-Specific Geroprotective Effects in Caenorhabditis elegans
Source: Int J Mol Sci. 2025 Nov 20;26(22):11205. doi: 10.3390/ijms262211205 (PMC12652852; doi:10.3390/ijms262211205)
Supplement: Supplementary file 1 [file ijms-26-11205-s001.zip › ijms-3950094-supplementary.pdf]

Supplementary figure

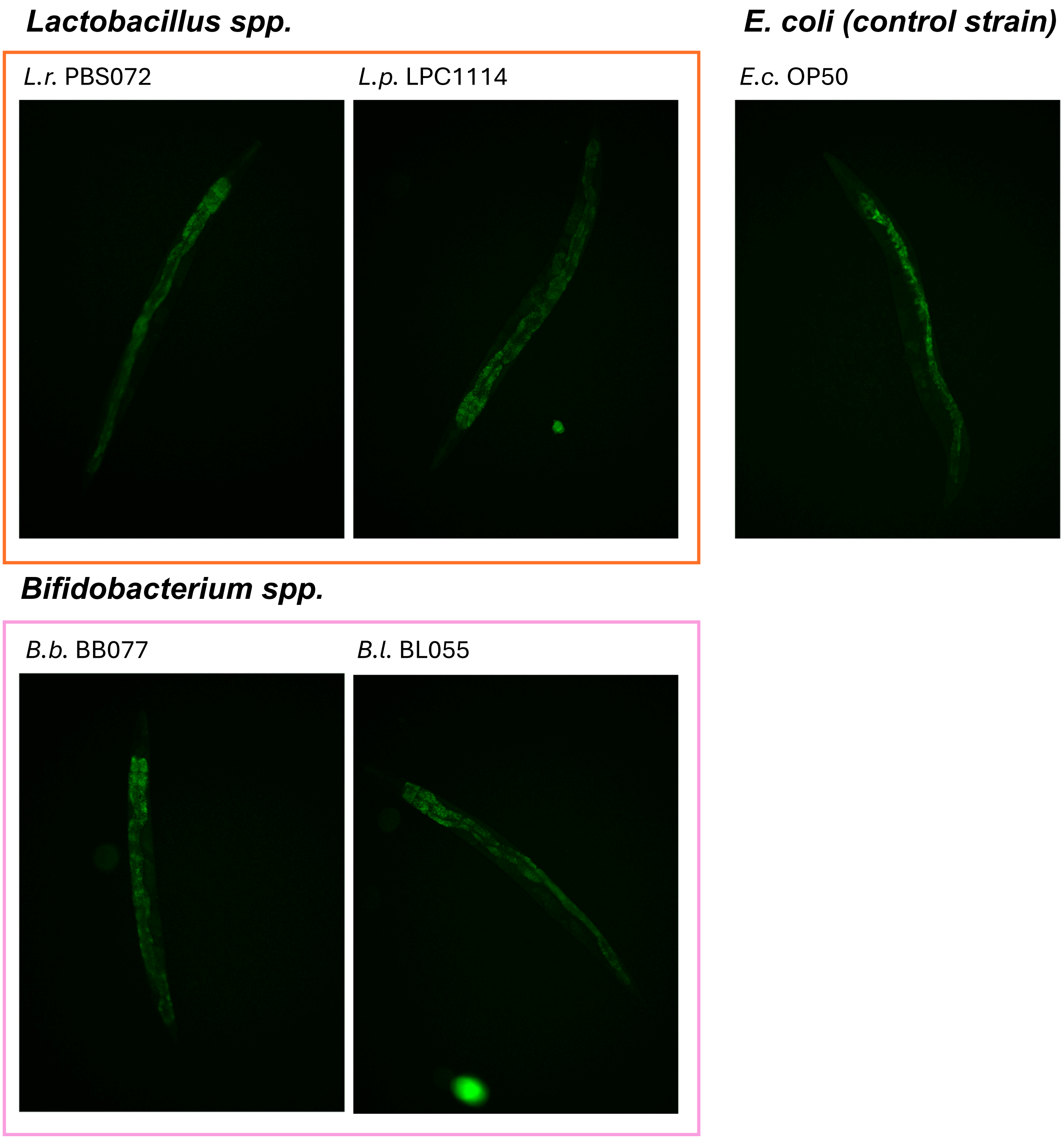

Figure S1: Representative images of lipofuscin in nematodes treated with probiotics from 1-day to 11-day of adulthood. See material and methods for experimental details.
